# Design and optimization of haze prediction model based on particle swarm optimization algorithm and graphics processor

**DOI:** 10.1038/s41598-024-60486-9

**Published:** 2024-04-26

**Authors:** Zuhan Liu, Kexin Zhao, Xuehu Liu, Huan Xu

**Affiliations:** School of Information Engineering, Nanchang Institute of Technology, Nanchang, 330099 China

**Keywords:** Haze prediction, Support vector regression, Parallel computing, Graphics Processing Unit, Environmental sciences, Mathematics and computing

## Abstract

With the rapid expansion of industrialization and urbanization, fine Particulate Matter (PM_2.5_) pollution has escalated into a major global environmental crisis. This pollution severely affects human health and ecosystem stability. Accurately predicting PM_2.5_ levels is essential. However, air quality forecasting currently faces challenges in processing vast data and enhancing model accuracy. Deep learning models are widely applied for their superior learning and fitting abilities in haze prediction. Yet, they are limited by optimization challenges, long training periods, high data quality needs, and a tendency towards overfitting. Furthermore, the complex internal structures and mechanisms of these models complicate the understanding of haze formation. In contrast, traditional Support Vector Regression (SVR) methods perform well with complex non-linear data but struggle with increased data volumes. To address this, we developed CUDA-based code to optimize SVR algorithm efficiency. We also combined SVR with Genetic Algorithms (GA), Sparrow Search Algorithm (SSA), and Particle Swarm Optimization (PSO) to identify the optimal haze prediction model. Our results demonstrate that the model combining intelligent algorithms with Central Processing Unit-raphics Processing Unit (CPU-GPU) heterogeneous parallel computing significantly outpaces the PSO-SVR model in training speed. It achieves a computation time that is 6.21–35.34 times faster. Compared to other models, the Particle Swarm Optimization-Central Processing Unit-Graphics Processing Unit-Support Vector Regression (PSO-CPU-GPU-SVR) model stands out in haze prediction, offering substantial speed improvements and enhanced stability and reliability while maintaining high accuracy. This breakthrough not only advances the efficiency and accuracy of haze prediction but also provides valuable insights for real-time air quality monitoring and decision-making.

## Introduction

Air quality has always been a major issue for human survival and development. With the progress of society, heavy industry, automobile manufacturing and other industries are constantly developing. This has promoted the process of urbanization but also led to a sharp increase in the content of various suspended particulate matter in the atmosphere. The problem of smog pollution has become increasingly prominent. Pollutants that form haze include gaseous pollutants such as SO_2_, NO_2_, CO, O_3_, and inhalable suspended particulate matter. According to the diameter of suspended particulate matter, it can be divided into PM_2.5_^1^, PM_10_ and PM_100_. Compared to other air pollutants, PM_2.5_ and other respirable particles not only have a serious impact on the quality of the atmosphere but also pose a significant threat to human health^2–4^. In Beijing, for example, there were 46 days of severe haze in 2015 alone. On average, severe polluted weather occurs every ten days. Therefore, in the case of frequent haze pollution events, scientific and effective PM_2.5_ prediction and early warning are very necessary. It helps to adopt timely and effective response methods to reduce the impact of smog pollution^5–7^.

In the field of haze pollution prediction, technologies like Recurrent Neural Networks (RNNs), Convolutional Neural Networks (CNNs), and Deep Belief Networks (DBNs) demonstrate significant adaptability, learning capacity, and strong model fitting ability^8^. However, the deployment of neural networks has its challenges. The intricacies of model tuning and optimization are non-trivial, often necessitating extensive computational resources and time^9,10^. Furthermore, their propensity for overfitting and the critical dependence on high-quality data can compromise prediction accuracy. These models' “black box” nature presents significant interpretative challenges by obscuring the causal mechanisms behind haze formation. This opacity impedes scientific inquiry into atmospheric quality. Compared to other methods, machine learning excels at analyzing numerous environmental factors and nonlinear relationships in haze prediction. The models are noted for their simplicity and the ease with which their results can be understood and explained. A recent study introduced a Support Vector Regression (SVR) model to estimate hourly atmospheric pollution concentrations. The findings indicate that SVR achieves high predictive accuracy^11^. Balogun et al.^12^ reported that an array of SVR-based models has been crafted for landslide prediction in western Serbia. Furthermore, still some scholars employed a hybrid SVR model to estimate crop transpiration, achieving a root mean square error of 0.21687, underscoring the model's high precision^13^. Karimipour et al.^14^ applied SVR to forecast the thermal conductivity of nanofluids, taking into account the effects of temperature and nanoparticle volume fraction. Their findings demonstrated that SVR possesses a robust resistance to overfitting, ensuring reliable predictions even with complex datasets.

Larger datasets present new challenges for traditional SVR in estimation and prediction. To enhance haze forecasting capabilities, this study developed specialized Compute Unified Device Architecture (CUDA) code for efficient execution of the SVR algorithm. Utilizing the MATLAB Executable (MEX) interface, we seamlessly integrated our custom CUDA implementation with the MATLAB environment. The paper employs a heterogeneous parallel computing architecture that leverages computational resources of both Central Processing Units (CPUs) and Graphics Processing Units (GPUs). This architecture offers efficient computation for large-scale datasets of environmental, meteorological, and pollutant emission data. Under this framework, SVR, with its lower complexity, limited parameter count, and simplified tuning process, demonstrates a significant acceleration advantage when handling large datasets and remains effective on smaller ones. Compared to deep neural networks, SVR does not require extensive datasets to prevent overfitting nor excessive time and resources for training. It is better suited for structured meteorological data, and the heterogeneous parallel architecture further enhances its computational efficiency and resource conservation in haze pollution prediction. However, the performance of SVR is heavily contingent upon parameter optimization. Population intelligence algorithms have emerged as a response to the computational demands and data quality requirements inherent in complex problem-solving. These algorithms offer a novel computational framework that is robust to hyperparameter selection, effectively navigating the challenges presented. Renowned instances encompass particle swarm optimization (PSO)^15^, ant colony optimization (ACO)^16^, immune algorithm (IA)^17^ and genetic algorithm (GA)^18^. The fusion of population intelligence and machine learning has been a focal point of recent research. For example, Feng et al*.*^19^ employed PSO to fine-tune support vector machines (SVM) for hydrological forecasting. Their approach demonstrated superior predictive accuracy when compared to conventional models such as artificial neural networks (ANN) and extreme learning machines (ELM). Bezdan et al*.*^20^ innovated a hybrid population intelligence approach by enhancing the fruit fly algorithm (FFA) with the search dynamics of the firefly algorithm (FA) and opposites-based learning (OBL). This approach was applied to optimize the k-means algorithm for text clustering, yielding superior results. Mallick et al*.*^21^ explored various hybrid models, including PSO-ANN, PSO-Random Forest (RF), PSO-Radial Basis Function (RBF), PSO-REP Tree and PSO-M5P to project climate change impacts in Saudi Arabia's Asir Basin. Their studies corroborated the PSO-RF model's exceptional predictive strength among the proposed combinations. The adaptability and excellent scalability of the PSO algorithm have been demonstrated in the aforementioned studies.

The studies indirectly indicate that ensemble machine learning models exhibit greater interpretability and explainability, enhanced robustness, and superior capability to avoid overfitting compared to deep learning. Building upon this foundation, this research introduces the Particle Swarm Optimization and CPU-GPU heterogeneous parallel Support Vector Regression model (PSO-CPU-GPU-SVR). Furthermore, we have integrated SVR with Genetic Algorithm (GA) and Sparrow Search Algorithm (SSA). By comparing the PSO-SVR, GA-CPU-GPU-SVR, and SSA-CPU-GPU-SVR haze prediction models on a haze dataset, we seek to identify the optimal model for haze forecasting. The experimental results suggest that the proposed PSO-CPU-GPU-SVR model performs well. It yields predictions with minimal error compared to actual values, meeting the precision requirements of the model and exhibiting impressive acceleration performance. To our knowledge, no prior work has applied a regression model that synergistically integrates particle swarm optimization with parallelized support vector mechanisms for air quality prediction. This indicates that the findings of this paper offer valuable insights for research in the field of haze forecasting.

## Methods

In the field of haze prediction, selecting an appropriate model is crucial for enhancing forecast accuracy. To this end, we developed optimized code based on the CUDA and designed a CPU-GPU heterogeneous parallel processing architecture for the SVR model, termed CPU-GPU-SVR. By distributing key computational steps of SVR across different processors for parallel execution, we significantly accelerated the model's training and prediction processes. Moreover, to precisely select parameters within the SVR model, we employed and enhanced the PSO algorithm for more effective service in SVR parameter optimization. The integration of the improved PSO algorithm with CPU-GPU-SVR not only substantially increased the model's prediction accuracy but also ensured the efficiency of processing large-scale data. Further, to validate the effectiveness of our proposed model and explore the optimal solution for haze prediction, we combined PSO, GA, and SSA with the CPU-GPU-SVR model in a series of comparative experiments. We aim to introduce an efficient and accurate new tool to the field of haze prediction through this approach. Subsequent sections of this paper will detail the implementation mechanism of heterogeneous parallel SVR, the optimization process of the PSO algorithm, and their specific application in conjunction with SVR. This research holds significant importance for real-time haze monitoring and rapid response measures, contributing to the reduction of haze's impact on the environment and public health.

### CPU-GPU heterogeneous parallelism approach

**Figure 1:**
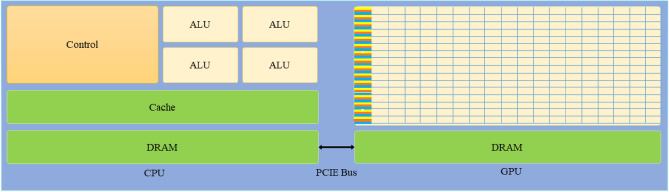
GPU and CPU internal architecture.

**Figure 2:**
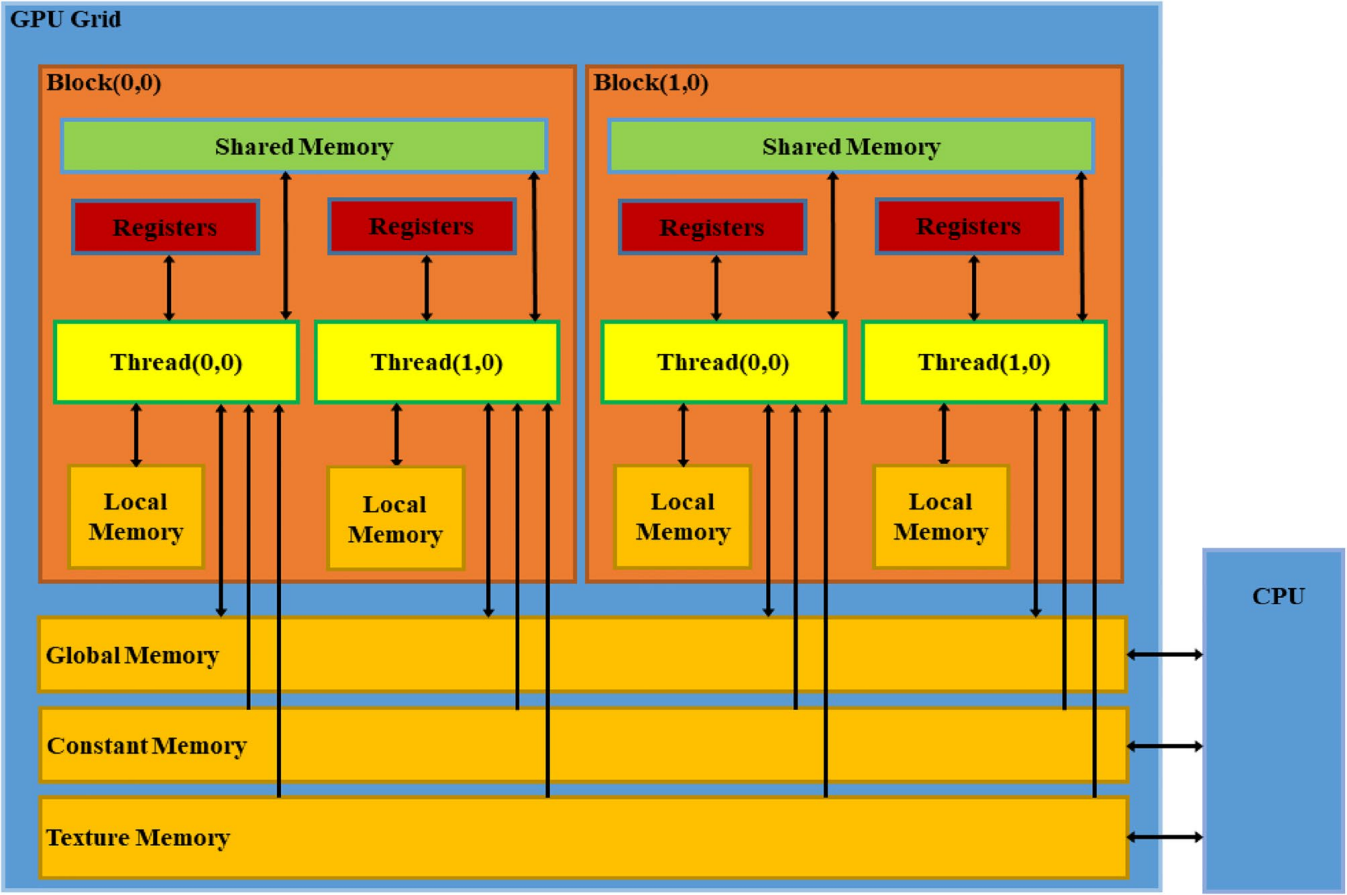
CUDA memory structure.

Compute Unified Device Architecture (CUDA) is a parallel computing architecture based on the graphics processing unit (GPU) introduced by NVIDIA in 2006, whose internal architecture is shown in Fig. 1. Nevertheless, the number of computation units within the GPU is much higher than the number of logical control units. So, under the CUDA architecture, a simple data computation model is first constructed to avoid complex instruction flow control. Then create a large-scale threaded computing model, where the CPU is responsible for complex logic processing and system scheduling, and the GPU is responsible for massively parallel computing. CPUs and GPUs play their respective strengths in collaborative computing. As the CPU and GPU have different storage spaces, CUDA provides different levels of memory to enable threads to run independently on the GPU device (see Fig. 2). During parallel program execution, CUDA threads may access data from multiple memory spaces. Each thread has private local memory (Local Memory) that exists only for the lifetime of that thread. Each thread block has Shared Memory that is visible to all threads within the block and has the same lifecycle as the thread block. All threads have access to the same Global Memory. Global Memory is allocated and released by the host side to initialize the data that will be processed by the GPU. Two other types of memory are read-only and accessible by all threads, Constant and Texture Memory. Constant memory is generally used to cache values that are shared by all functions, and texture memory is used for some of the texture operations provided by the hardware. The global, constant and texture memory spaces are optimized for different memory uses.

**Figure 3:**
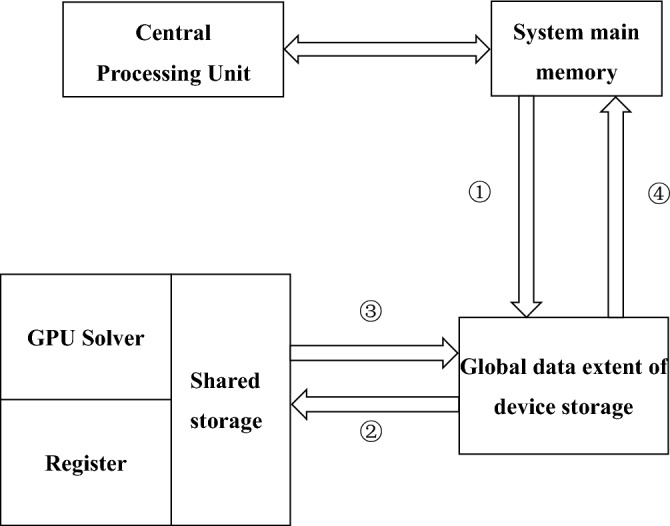
CPU-GPU heterogeneous parallel processing flow.

The CUDA programming model uses the CPU as the Host for logical tasks and data read-in and output operations, and the GPU as the Device for data-intensive and highly parallel tasks. In this case, the main program is controlled by the CPU. When a GPU kernel function is to be executed, the CPU first copies the required data from the system host memory to the GPU global memory. Then multiple GPU threads are stimulated to run the kernel function. When the GPU computation is complete, the resulting result is passed back to the system’s main memory via the global memory and handed over to the CPU for further processing (see Fig. 3).

### Support vector regression models

The theory of Support Vector Machine (SVM) was proposed by Vapnik and Corina Cortes in the late twentieth century. Then Chang and Kyung proposed SVR models for regression problems, which have received increasing attention in regression estimation and non-linear problem-solving. SVR is the mapping of input quantities to a high-dimensional plane, which is transformed into a linear regression relationship. Assume a regression function ƒ(*x*) with a training sample set of (*x*_*i,*_*y*_*i*_) *i* = 1,2,…,*m*. SVR uses a linear equation to predict the target value, i.e.

1 $$f(x) = w^{T} \varphi (x) + b{\kern 1pt} {\kern 1pt} {\kern 1pt} {\kern 1pt} w \in R^{n} {\kern 1pt} {\kern 1pt} {\kern 1pt} {\kern 1pt} b \in R$$

where ƒ(*x*), ***w*** and ***b*** are the output, the weight vector and the threshold respectively. *φ*(*x*) is the high-dimensional actual input vector. The weight vectors ***w*** and ***b*** are calculated by minimizing the risk function. The inference process for minimizing risk is as in Eqs. (2)–(5).

2 $$k(f) = C\sum\limits_{i = 1}^{m} {L_{\varepsilon } } (y_{i,} f_{i} ) + \frac{1}{2}||w||^{2}$$

3 $$L_{\varepsilon } (y_{i,} f_{i} ) = \left\{ {\begin{array}{*{20}l} {0,} \hfill & {if\;|y_{i} - f_{i} |{\kern 1pt} {\kern 1pt} \le \varepsilon } \hfill \\ {|y_{i} - f_{i} | - \varepsilon ,} \hfill & {otherwise} \hfill \\ \end{array} } \right.$$

In Eq. (2), the first term uses an insensitivity function *ɛ* to represent the deviation between the actual and regressed values. The second term is used to estimate the ambiguity of the function. *C* is the penalty parameter, making a compromise between the two. In the actual problem, it is difficult to determine *ɛ* exactly. To ensure that most of the data points are within *ɛ*, the slack variables *ξ*_*i*_ and *ξ*_i_^*^ are introduced and Eq. (2) is transformed into the following form.

4 $$\begin{aligned} k(w) & = \min \frac{1}{2}||w||^{2} + C\sum\limits_{i = 1}^{m} {(\xi_{i} } + \xi_{i}^{*} ) \\ & \quad s.t\;\left\{ {\begin{array}{*{20}l} {f(x_{i} ) - y_{i} \le \varepsilon + \xi_{i} } \hfill \\ {y_{i} - f(x_{i} ) \le \varepsilon + \xi_{i}^{*} } \hfill \\ {\xi {\kern 1pt} > {\kern 1pt} 0{\kern 1pt} {\kern 1pt} \xi_{i}^{*} {\kern 1pt} > {\kern 1pt} 0{\kern 1pt} {\kern 1pt} i{\kern 1pt} = {\kern 1pt} 1{\kern 1pt} ,...,{\kern 1pt} m} \hfill \\ \end{array} } \right. \\ \end{aligned}$$

Considering the non-linear regression case, the mapping of the feature space and the introduction of the LaGrange function, Eq. (1) is transformed to (5)

5 $$f = \sum\limits_{i = 1}^{m} {(\alpha_{i} - \alpha_{i}^{*} )} k(x_{i,} x) + b$$

### Sequential minimal optimization

The indicator variable f associated with the training sample in the SMO algorithm is defined as follows.

6 $$f = \sum\limits_{i = 1}^{m} {\alpha_{i} } y_{i} k(x,x_{i} ) - y$$

where *α*_*i*_ = (*α*_i_^*^−α_*i*_), SMO algorithm solves the SVR in the following steps.

Find two training samples *x*_*u*_ and *x*_*l*_, which together form a working set^22–24^. The indicator variables associated with them *f*_*u*_ and *f*_*l*_, satisfy a maximum and minimum condition, respectively, where the subscripts *u* and *l* are updated with the formulas.
  7 $$u = \arg \min_{i} \left\{ {f_{i} |i \in I_{u} } \right\}$$
  8 $$l = \arg \max_{i} \left\{ {\frac{{(f_{u} - f_{i} )^{2} }}{{\eta_{i} }}|f_{u} < f_{i} {\kern 1pt} {\kern 1pt} {\kern 1pt} {\kern 1pt} i \in I_{l} } \right\}$$
  *f*_*u*_ and *f*_*l*_ are found using Eqs. (7) and (8). *I*_*u*_ and *I*_*l*_ are defined as follows.
  9 $$\begin{aligned} I_{u} & = {\kern 1pt} I_{1} {\kern 1pt} \cup {\kern 1pt} I_{2} {\kern 1pt} \cup {\kern 1pt} I_{3} {\kern 1pt} {\kern 1pt} {\kern 1pt} {\kern 1pt} I_{l} {\kern 1pt} = {\kern 1pt} I_{1} {\kern 1pt} \cup {\kern 1pt} I_{4} {\kern 1pt} \cup {\kern 1pt} I_{5} \\ I_{1} & = \{ i|x_{i} {\kern 1pt} \in {\kern 1pt} X{\kern 1pt} {\kern 1pt} {\kern 1pt} 0{\kern 1pt} < {\kern 1pt} \alpha_{i} {\kern 1pt} < {\kern 1pt} C\} \\ I_{2} & = i|x_{i} {\kern 1pt} \in {\kern 1pt} X{\kern 1pt} {\kern 1pt} y_{i} {\kern 1pt} = {\kern 1pt} \varepsilon + \xi_{i} {\kern 1pt} {\kern 1pt} \alpha_{i} {\kern 1pt} = 0\} \\ I_{3} & = i|x_{i} {\kern 1pt} \in {\kern 1pt} X{\kern 1pt} {\kern 1pt} y_{i} {\kern 1pt} = {\kern 1pt} - {\kern 1pt} \varepsilon {\kern 1pt} + {\kern 1pt} \xi_{i}^{*} \alpha_{i} {\kern 1pt} = {\kern 1pt} C\} \\ I_{4} & = i|x_{i} {\kern 1pt} \in {\kern 1pt} X{\kern 1pt} {\kern 1pt} y_{i} {\kern 1pt} = {\kern 1pt} \varepsilon + \xi_{i} {\kern 1pt} {\kern 1pt} \alpha_{i} {\kern 1pt} = {\kern 1pt} C\} \\ I_{5} & = \{ i|x_{i} {\kern 1pt} \in {\kern 1pt} X{\kern 1pt} {\kern 1pt} y_{i} {\kern 1pt} = {\kern 1pt} - {\kern 1pt} \varepsilon {\kern 1pt} + {\kern 1pt} \xi_{i}^{*} {\kern 1pt} {\kern 1pt} \alpha_{i} {\kern 1pt} = {\kern 1pt} 0\} \\ \end{aligned}$$
  *Il* contains all free support vectors*, I2* and *I5* contain all non-support vectors, and *I3* and *I4* contain all support vectors that are inside the boundary.
(2) Update the weights *α*_*u*_ and *α*_*l*_ for* x*_*u*_ and* x*_*l*_.
  10 $$\alpha^{\prime}_{u} = \alpha_{u} + y_{l} y_{u} (\alpha_{l} - \alpha^{\prime}_{l} )$$
  11 $$\alpha^{\prime}_{l} = \alpha_{l} + \frac{{y_{l} (f_{u} - f_{l} )}}{\eta }$$
  The range of values for $\alpha^{\prime}_{u}$ and $\alpha^{\prime}_{l}$ is [0*, C*].
Update the formula for the indicator variable for all training samples as follows.
  12 $$f^{\prime}_{i} = f_{i} + \left( {a^{\prime}_{u} - a_{u} } \right)y_{u} k\left( {x_{u} ,x_{i} } \right) + \, \left( {a^{\prime}_{l} - a_{l} } \right)y_{l} k\left( {x_{l} ,x_{i} } \right)$$
  The SMO algorithm repeats these three steps until the termination condition is met.
  13 $$f_{u} = \min \{ f_{i} |i \in I_{u} \} \ge f_{\max }$$
  14 $$f_{\max } = \max \{ f_{i} |i \in I_{l} \}$$

### Particle swarm optimization algorithms

In this study, we chose the PSO algorithm over other swarm intelligence algorithms to accelerate the parameter optimization of SVR. This decision was based on several considerations. Known for its simple structure and fewer parameters to adjust, the PSO algorithm is easy to understand and implement^25^. Furthermore, the independent particle search mechanism of PSO naturally lends itself to parallel computing, which is particularly beneficial for exploiting the high parallel performance of GPUs. The update and evaluation of each particle can be executed in parallel, significantly enhancing computational efficiency. Additionally, PSO typically achieves faster convergence rates, crucial for time-constrained scenarios. Compared to GA and SSA, PSO requires less memory, offering a clear advantage in resource-limited GPU environments. Although PSO may risk getting trapped in local optima, it has proven its capability to find global optima across various test problems, making it an effective choice for accelerating SVR parameter optimization.

PSO are referred to as particles, and the population consists of *N* particles initialized in a *D* search space. During the search process, each particle *i* is represented by two vectors: a velocity vector *V*_*i*_ = [*v*_*i1*_*,v*_*i2*_*,…,v*_*iD*_] and a position vector* X*_*i*_ = [*X*_*i1*_*,X*_*i2*_*,…,X*_*iD*_]. Each particle updates its velocity and position in space using its personal best position *P*_*id,pbest*_ = [*P*_*i1*_*,P*_*i2*_*,…,P*_*iD*_] and the global best position* P*_*d,gbest*_ = [*P*_*1,gbest*_*,P*_*2,gbest*_*,…,P*_*D,gbest*_]. For simplicity, *P*_*bi*_ denotes* P*_*id,pbes*_, and *P*_*g*_ represents* P*_*d,gbest*_. The update formulas for *X*_*i*_ and *V*_*i*_ are shown in Eqs. (15) and (16):

15 $$Pb_{id}^{t + 1} = \left\{ {\begin{array}{*{20}l} {X_{id}^{t + 1} ,} \hfill & {f(X_{id}^{t + 1} ) \le f(Pb_{id}^{t} )} \hfill \\ {Pb_{id}^{t} ,} \hfill & {f(X_{id}^{t + 1} ) > f(Pb_{id}^{t} )} \hfill \\ \end{array} } \right.$$

16 $$Pg_{d}^{t + 1} = \left\{ {\begin{array}{*{20}l} {Pg_{d}^{t} ,} \hfill & {f(Pg_{d}^{t} ) \le \mathop {\min }\limits_{i} (f(Pb_{id}^{t + 1} ))} \hfill \\ {Pb_{id}^{t + 1} ,} \hfill & {f(Pg_{d}^{t} ) > \mathop {\min }\limits_{i} (f(Pb_{id}^{t + 1} ))} \hfill \\ \end{array} } \right.$$

To accelerate the convergence speed and enhance the global search capability of the PSO algorithm, we introduced an inertia weight *w*. This modification transforms the particle velocity update equation as follows:

17 $$V_{id}^{{{\text{t}} + 1}} = w *V_{id}^{t} + c_{1} *r_{1} * [Pb_{id}^{t} - X_{id}^{t} ] + c_{2} *r_{2} *[Pg_{d}^{t} - X_{id}^{t} ]$$

In PSO, the setting of the inertia weight is crucial for the search capability. A larger inertia weight promotes global exploration, while a smaller one favors local exploitation. However, a fixed inertia weight may constrain the algorithm's local search efficiency in later stages, leading to slow convergence and an increased risk of becoming trapped in local optima. To address the nonlinearity and multimodality of inversion problems, we innovatively propose an adaptive inertia weight formula. This formula dynamically adjusts the inertia weight based on the difference between each particle's fitness and the worst fitness in the initial swarm. This allows the PSO to more flexibly balance between global exploration and local exploitation, potentially improving convergence speed and reducing the risk of local optima entrapment. The updated formula is presented below.

18 $$w_{i} = w_{\max } - (w_{\max } - w_{\min } )\frac{{f_{i}^{t} - f_{worst} }}{{f_{best} - f_{worst} }}$$

In the formula, *w*_max_ and *w*_min_ represent the maximum and minimum inertia weights, respectively. In this study, they are set to 0.9 and 0.4. *f*_*worst*_ and *f*_*best*_ denote the worst and best fitness of the initial swarm, respectively. The term *f*_*i*_^*t*^ indicates the fitness of the *i*-th particle at the *t*-th iteration. The randomness of initial particle positions in PSO leads to significant differences between the initial best and worst fitness values. Our research incorporates this difference into the calculation formula, effectively differentiating the inertia weights of particles with varying fitness levels. This enhances the global search capability in the early stages of the algorithm. As fitness improves, the inertia weight of particles decreases, slowing their velocity to facilitate meticulous local search and prevent overshooting the optimal solution.

### Haze prediction method based on particle swarm algorithm and CPU-GPU heterogeneous parallel SVR.

For SVR training and prediction, LIBSVM^26^ is a widely chosen package for SVR computing and is used by many AI and machine learning frameworks as the underlying SVR computing implementation. However, the computational cost of basic SVR training and analysis is high for large and complex problems. LIBSVM is optimally accelerated for early single-core CPUs and provides limited parallelization support. However, with the dramatic increase in the amount of raw data and problem size, the training and prediction speed of LIBSVM is often insufficient to meet the actual algorithm debugging and application needs.

Therefore, how to improve the training and prediction speed of SVR is a problem that needs to be considered in this algorithm^27,28^. To address the computational challenges of SVR when processing large-scale data, this study developed specialized CUDA code for efficient execution of the SVR algorithm. We seamlessly integrated our custom CUDA implementation with the MATLAB environment using MATLAB's external interface capabilities. The paper employs a heterogeneous parallel computing architecture that combines the computational resources of CPUs and GPUs to optimize the computational workflow of the SVR algorithm. This approach significantly enhances the algorithm's computational efficiency while maintaining the convenience of scientific computing and data analysis within MATLAB. The architecture leverages the CPU for high-level control of the algorithm and execution of complex operations. It utilizes the GPU's parallel processing capabilities to accelerate kernel function computations and matrix operations within the algorithm. This parallel structure markedly improves computational efficiency, particularly in time series forecasting involving large datasets. The GPU can process thousands of computational threads in parallel, thus reducing the training and prediction time of the SVR model. Moreover, compared to traditional CPU architectures, the heterogeneous parallel structure demonstrates clear advantages in reducing energy consumption and hardware costs. With advancements in GPU technology, the architecture enables the SVR model to scale to larger datasets, thereby enhancing prediction accuracy and the model's generalization capability. On the optimization front, the adaptability of the architecture ensures that CPU and GPU computational resources can be dynamically allocated according to the specific demands of the task. Therefore, adopting the CPU-GPU heterogeneous parallel computing architecture not only drives a computational efficiency revolution for SVR in time series prediction tasks but also paves the way for innovative applications such as real-time or near-real-time forecasting. It exhibits significant practical value in critical application areas like meteorological forecasting.

#### CPU-GPU heterogeneous parallel SMO-based model

Based on the CUDA programming platform, this paper implements the SVR algorithm by using the CPU-GPU heterogeneous parallel method and mainly uses two parallel strategies, vectorization, and parallel protocol. Vectorization is a fundamental tool for parallelizing algorithms. In numerical optimization problems, many operations can be represented as vectors. By implementing vectorization operations in the GPU, reading multiple operands at once and performing the same operation on all operands at the same time can greatly reduce the time to complete vector operations. The parallel statute algorithm is also a basic parallelization tool. When many operands in the GPU need to perform the same operation, the parallel regulation algorithm starts multiple threads at the same time. Each thread picks two operands to perform the corresponding operation. All threads execute multiple turns, halving the number of threads per execution, and finally completing all operands. Therefore, we find the vectorizable part of the optimization algorithm or convert scalar operations to vector operations and normalization operations to improve the calculation speed.

The SMO algorithm solves the SVR process as follows.
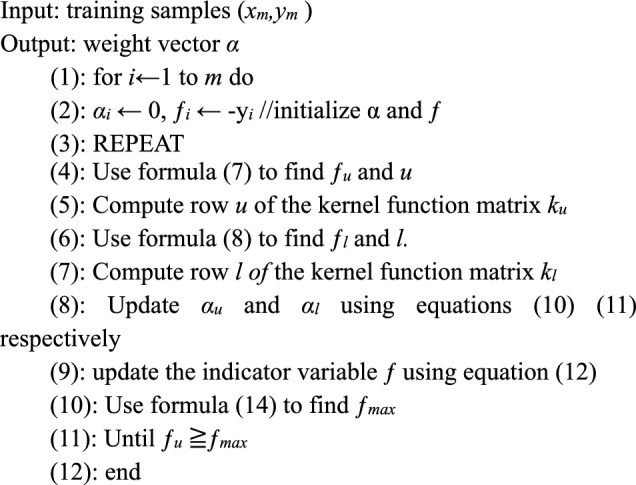


The main process for parallelizing the improved sequence minimization algorithm using vectorization and parallel statute strategies is as follows.

1. Since the GPU side is unable to start itself and input and output data, it relies on the CPU to configure the environment, transfer data, and start kernel functions.
2. Initialize α and matrix ƒ of steps 1–2 using vectorized assignment operations.
3. Parallelize step 4 with vectorization and parallel protocol operations. Specifically, the selection of the working set is implemented on the GPU i.e., finding the desired samples in the data set satisfying Eqs. (7) and (8). The CUDA has strict restrictions on communication and synchronization between blocks of processing threads, which makes the implementation of reduction operations such as finding the maximum value of a vector or sums very important. After initialization is complete, each block uses the statute algorithm to compute the local optimum. Their output is a vector of 64 candidate values, accompanied by indexes copied into CPU memory. Since the CPU has a greater efficiency advantage over the GPU in performing very small reductions, the CPU comes to perform the final sequential reduction. The same method can also be used to find *ƒ*_*l*_ and *l* in step 6 and *ƒ*_*max*_ in step 10.
4. In step 8, the *α*_*u*_ and *α*_*l*_ matrices are updated using Eqs. (10) and (11). *α*_*u*_ and *α*_*l*_ are shared by each computing process. Combined with the shared memory in the CUDA programming architecture, all data is read at once. It is then shared by threads within the block, reducing data exchange between global memory and threads and improving computational efficiency.
5. In step 9, there is no dependency between the dimensions of ƒ, and the GPU uses Eq. (12) to achieve a fully parallelized update of the ƒ matrix using a vectorization strategy to obtain a significant speed-up.
6. A conditional judgement is made at the CPU side for step 10 to decide whether the loop ends or not. If the loop ends, the obtained LaGrange factor and the values of *u* and *l* are transferred back to memory, and then the CPU side gives the *b* value and outputs the end of the decision model training.

#### Parallel implementation of working set selection

The procedure for calculating *l* and ƒ_***l***_ using the parallel statute method is as follows.

1. Before calculating *l* and ƒ_*l*_, ƒ has been calculated, that is, each thread calculates the value of a ƒ array. Store the value of ƒ in a ƒ array in shared memory and store the value of the subscript corresponding to ƒ, i.e., *l*, in an *l* array located in shared memory.
2. All threads in each block perform the statute operation on the maximum value and store the sub-result obtained from each block at the first address of the block.
3. Each block performs a data transfer operation, copying ***f*[0]** and ***l*[0]** from shared memory to the array ***GlocbalF*[0]** and the array ***GlobalI*[0]** arrays located in global memory.
4. Perform a max-stat operation on the array ***GlocbalF*[0]** and the array ***GlobalI*[0]** to obtain the values of *l* and ƒ_*l*_.

#### Parallel operations on kernel function matrices

Choosing the right kernel function and parameters can improve prediction accuracy and reduce the influence of noise. The radial basis (RBF) kernel function has the advantages of strong learning ability, strong adaptability in high and low dimensions, wide convergence range, good performance stability, and few adjustment parameters. Therefore, this paper discusses the parameter optimizations of the SVR kernel function based on the RBF kernel function.

19 $$k(x \cdot x_{i} ) = \exp \left( { - \frac{{||x - x_{i} ||^{2} }}{{\sigma^{2} }}} \right)$$

The main parameters affecting SVR performance are penalty factor *C* and the radial basis radius of the RBF kernel function *σ*. The smaller the *C* and the smaller the error loss on the model, which result in a decrease in the accuracy of training the SVR model. The larger *C* and the greater the penalty for error and the likelihood of overfitting the model. *σ* has a direct impact on the formation of the regression hyperplane. When *σ* → 0, the decision function of the support vector machine will be close to a constant, which results in lower regression accuracy for both the training and prediction samples. When *σ* → ∞, although the regression accuracy of the training samples is high, the generalization ability is poor, i.e., the prediction accuracy of the test samples is low. The *||x − x*_*i*_*||*^2^ terms expand as follows.

20 $$\left\| {x - x_{i} } \right\|^{2} = \left( {x_{0}^{2} + x_{i0}^{2} - 2x_{0} x_{i0} } \right) + \left( {x_{1}^{2} + x_{i1}^{2} - 2x_{1} x_{i1} } \right) + \cdots + \left( {x_{m}^{2} + x_{im}^{2} - 2x_{m} x_{im} } \right)$$

The kernel function operation is the most complex and time-consuming part of the SMO algorithm, and the kernel function values of the current two sample data need to be calculated in each iteration. Calculating each element in the kernel function matrix requires only two corresponding data corresponding to it *x* and *x*_*i*_ and no additional sample data. Because there is no correlation between any two different elements in the matrix, this satisfies the conditions for parallelization.

The parallelization of the kernel function matrix is calculated in a similar way to the matrix–vector multiplication operation. The set of data samples is formed into a matrix of the form. Matrix ***A*** consists of m sample vectors of ***1* × *k*** dimensions, matrix ***B*** is a transpose of matrix ***A***, and the kernel function matrix is matrix ***C*** of ***m* × *m*** dimensions. Each row of the matrix has m kernel functions to be computed, with a minimum time complexity of *O*(*mk*). The kernel function corresponding to the line *u* example is as follows.

21 $$k_{u} = k(x_{u} ,x_{1} ),k(x_{u} ,x_{2} ),...,k(x_{u} ,x_{n} )$$

Based on the above calculation method for the kernel function matrix, the parallel calculation of the kernel function is implemented using the GPU as follows.

1. The initialization of the GPU is first implemented on the CPU side. The *cudaMalloc* function is used to allocate storage space on the video memory to hold the sample data and the kernel function matrix. And then the *cudaMemcpy* function is used to copy the training samples from memory to video memory.
2. After the CPU starts the kernel function, each thread in the GPU reads the row of matrix ***A*** and the column of matrix ***B*** and then executes the corresponding instructions to calculate the value of the RBF kernel function, i.e. The value *k*(*x*_*i*_,*y*_*i*_) in matrix ***C***. During this process, each row of matrix ***A*** is read repeatedly. To reduce the number of reads, each element of matrix ***A*** is placed in shared memory with faster access, which improves program performance.
3. Use the *_syncthreads* function to perform thread synchronization operations.
4. Finally, use the *cudaMemcpy* function again to copy the computed kernel function matrix from the video memory back to memory, freeing up GPU memory space.

#### PSO-based optimization of CPU-GPU heterogeneous parallel SVR

Hyper-parameters can largely affect the generalization ability of the CPU-GPU heterogeneous parallel SVR model. Namely, the penalty factor *C* and the kernel function *σ*. It is difficult to determine the appropriate values of the parameters through a priori knowledge and the process of manually tuning the parameters is very time-consuming. To make the model evaluation more accurate and credible, the particle swarm optimization algorithm was used to find the optimal values for the *C* and *σ* parameters in Eqs. (2) and (15). The steps for parameter optimization of the CPU-GPU heterogeneous parallel SVR model using the particle swarm algorithm are as follows.

**Figure 4:**
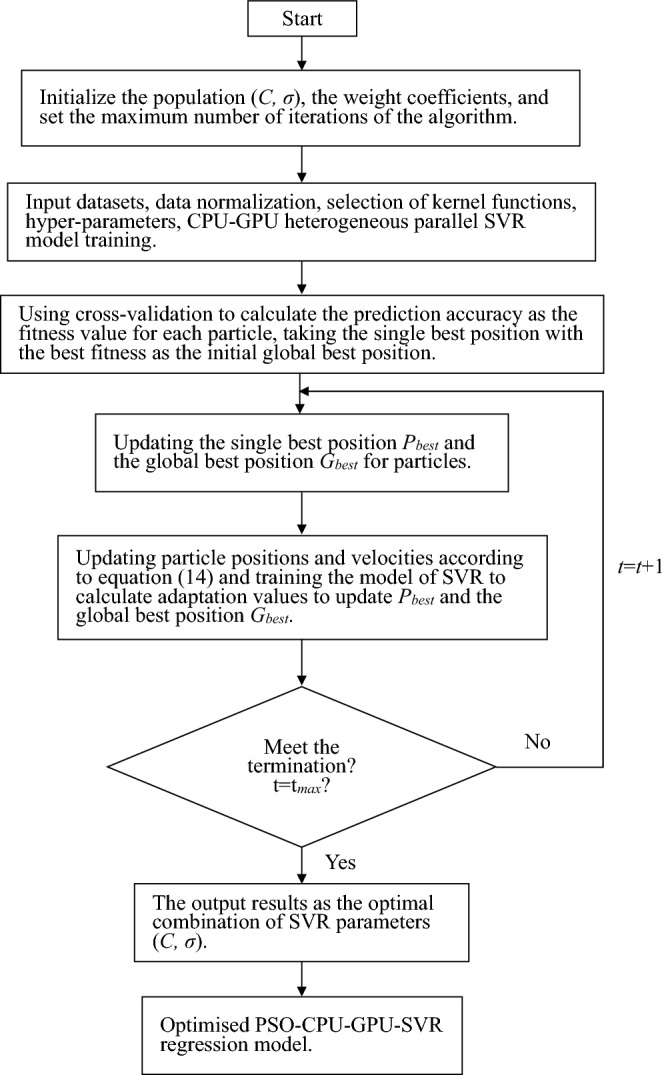
PSO-CPU-GPU-SVR flow chart.

1. Initialize the population (*C*, *σ*), set the weight coefficients *w* and set the maximum number of iterations of the algorithm *t*_*max*_.
2. Input datasets, data normalization, selection of kernel functions, hyper-parameters, CPU-GPU heterogeneous parallel SVR model training.
3. Using cross-validation to calculate the prediction accuracy as the fitness value for each particle, taking the single best position with the best fitness as the initial global best position.
4. Update the single best position *P*_*best*_ and the global best position *G*_*best*_ of the particle.
5. Update the particle positions and velocities according to Eq. (14) and train the model of the SVR to calculate the adaptation value update *P*_*best*_ and the global best position *G*_*best*_.
6. Determine if the termination condition is satisfied and the loop step is not satisfied ④–⑤. otherwise, the algorithm terminates and the output optimal solution is the optimal combination of parameters (*C*, *σ*) The PSO-CPU-GPU-SVR flowchart is shown in Fig. 4.

## Experiments

### Experimental design

The primary objective of this study is to verify the efficacy of the proposed CPU-GPU-SVR model, integrated with the PSO algorithm, in predicting PM_2.5_ concentrations. In addition, this research compares the performance differences between this model and the CPU-GPU-SVR model integrated with GA and SSA. We also evaluate its advantages in processing speed and accuracy relative to the traditional PSO-SVR model. To mitigate the influence of randomness inherent in heuristic algorithms and enhance the reliability of test results, we have standardized the population size and iteration number for all heuristic algorithms at 30 and 500, respectively. Consequently, all integrated models are run independently 30 times to ensure the attainment of the optimal values of the objective function across 30 independent runs. In terms of technical specifications, both the SVR model and the CPU-GPU-SVR model utilize the RBF kernel function, with the value range for ***C*** set between 0.01 and 100, and for ***σ***, between 0.001 and 100. All models employed fivefold cross-validation during the training process (*V* = 5). The detailed parameter settings for the experiment are meticulously outlined in Table 1.

**Table 1:** Parameter values for the competitor algorithms.

Algorithm name	Parameter setting
GA	*ggap* = 0.9, *px* = 0.7, *pm* = 0.7, *PRECI* = 20
PSO	*pop* = 30, *Maxiteration* = 500, *run* = 30, *w*_max=_0.9,w_min=_0.4,*c*1 = *c*2 = 1.2, *k* = 0.6
SSA	*pop* = 30, *Maxiteration* = 500, *run* = 30, *PD* = 70%, *ST* = 60%, *SD* = 20%

Models	Parameter setting
PSO-SVR	*ɛ* = 0.4, *V* = 5, *Max_iteration* = 100, run = 30
PSO-CPU-GPU-SVR	*c1* = *c2* = 1.2, *w*_max_ = 0.9, *w*_min_ = 0.4, *k* = 0.6, *ɛ* = 0.4, *Max_iteration* = 100, *pop* = 30, *run* = 30
GA-CPU-GPU-SVR	*ggap* = 0.9, *px* = 0.7, *pm* = 0.7, *PRECI* = 20, *ɛ* = 0.4, *Max_iteration* = 100, *pop* = 30, *run* = 30
SSA-CPU-GPU-SVR	*PD* = 70%, *ST* = 60%, *SD* = 20%, *ɛ* = 0.4, *Max_iteration* = 100, *pop* = 30, *run* = 30

To further explore the optimal model for haze prediction and better characterize the model's fit, this study introduces the Mean Absolute Percentage Error (MAPE), Root Mean Square Error (RMSE), and Coefficient of Determination (R^2^). Based on these metrics, we performed a statistical analysis of the discrepancies between the predicted results of several models and the ideal values. A smaller MAPE and RMSE indicate greater predictive accuracy of the model. The R^2^ primarily measures the fit of the model in regression analysis. Ranging between 0 and 1, it represents the percentage of the variance in the data that the model accounts for. An R^2^ of 1 signifies that the model perfectly fits the data. Conversely, an R^2^ of 0 implies that the model explains virtually none of the data's variance. The formula for calculating the R^2^ is as follows.

22 $$R^{2} = \frac{{\sum\nolimits_{i = 1}^{n} {(y_{i}^{*} - \overline{y}_{i} )^{2} } }}{{\sum\nolimits_{i = 1}^{n} {(y_{i} - \overline{y}_{i} )^{2} } }}$$

In the formula, *y*_i_^*^ represents the predicted value of the objective function, obtained through regression modeling. The term *y*_*i*_ denotes the actual value of the target variable for the sample. The experimental environment in this paper involves code written in a mixed software environment of MATLAB R2018b, Visual Studio 2013 and CUDA8.0. Compiled and run on a PC with the following parameters: 64-bit Windows 10 operating system, 11th Gen Intel(R) Core(TM) i7-11800H@2.30GHZ, 64 GB of RAM on board and an NVIDIA GeForce RTX 3070 GPU. In addition, none of the solving times includes file *I/O* and the parallelized computation times include all data transfers between CPU and GPU memory.

### Data sources and pre-processing

To evaluate the performance and generalization ability of our proposed model, we meticulously processed the experimental datasets. This ensures an increase in data scale for a thorough examination of the model's potential advantages for handling datasets of varying sizes.

#### Data sources

The datasets involved in the experiments are hourly Air Quality UCI, Beijing PM_2.5_ and Multi-Site Air-Quality data. Air pollution data is published on the regression database of the UCI machine learning database (https://archive.ics.uci.edu/ml/index.php). This dataset provides information on air quality in an Italian city from March 2004 to March 2005. PM_2.5_ concentration and air quality information in various areas of Beijing are from January 2010 to December 2014 and March 2013 to February 2017 respectively. The picture is the sampling distribution of haze data from March 2013 to February 2017 for several regions in Beijing of China.

The data set includes information on pollutants such as PM_2.5_, PM_10_, SO_2_, NO_2_, CO, and O_3_ and environmental information such as temperature, humidity, pressure, rainfall and wind speed. The types and meanings of the data are shown in Table 2.

**Table 2:** Data's types and meanings in the datasets.

Type	Sense	Type	Sense
No	Number ordinal	CO	CO concentration (ug/m^3^)
Year	Year ordinal	O_3_	O_3_ concentration (ug/m^3^)
Month	Month ordinal	TEMP	Temperature (℃)
Day	Day ordinal	PRES	Pressure (h/Pa)
Hour	Hour ordinal	DEWP	Dew Point Temperature
PM_2.5_	PM_2.5_ concentration (ug/m^3^)	RAIN	Precipitation (mm)
PM_10_	PM_10_ concentration (ug/m^3^)	WD	Wind Direction
SO_2_	SO_2_ concentration (ug/m^3^)	WSPM	Wind Speed (m/s)
NO_2_	NO_2_ concentration (ug/m^3^)	Station	Record the site

#### Data pre-processing

For the Beijing PM_2.5_ dataset, considering its data originates from a single monitoring site, we divided the dataset into two subsets of different sizes based on the length of the time series. Regarding the Beijing Multi-Site Air-Quality Data, it encompasses air quality observations from 12 monitoring sites in Beijing from March 2013 to February 2017. To orderly expand the data scale, we sliced the data in a 2:3:5 ratio based on the number of sites. In devising the dataset division strategy, this study first ensured an adequate scale of datasets to provide a solid testing ground for model evaluation. Given the voluminous nature of the datasets, we reserved 10% of the data as the test set, a proportion considered sufficient for an effective assessment of the model's generalization ability. Furthermore, we referred to the findings of References 29, whose research demonstrated outstanding performance on test sets of similar proportions. Therefore, combining the data volume and insights from previous studies, this paper adopts a 90:10 dataset division ratio, reserving 90% of the data for training and 10% for testing. We aim to achieve maximum training dataset utilization while ensuring ample test coverage.

During the process of selecting representative monitoring stations, we employed a greedy algorithm to ensure comprehensive coverage of the study area by the chosen sites. Given the central location of the Olympic Center in Beijing, we designated it as the initial representative station. Subsequently, we iteratively selected stations that were geographically furthest from the existing set until we obtained the required number of representative sites. This method aims to maximize the spatial distribution distance between representative sites. It ensures a broad and balanced coverage of air quality conditions across different areas of Beijing. This approach provides a diverse data foundation for the model, enabling a comprehensive assessment of its performance and generalization capabilities. Moreover, the original data contained some missing values. For missing data sequences not exceeding five hours in length, we employed linear interpolation for supplementation. Linear interpolation estimates missing data by inserting a new data point between two known values, assuming a linear change between data points. This method is particularly effective for time-series data. However, for longer periods of missing data, linear interpolation may introduce significant errors due to the extended duration of absence. Therefore, we opted to discard these portions of the data. After the aforementioned processing, the data used in this study is highly reliable. To enhance the efficiency of data processing and facilitate fair comparison across different datasets, all experimental data in this paper was normalized and mapped to the [0,1] interval. This normalization reduces scale differences between data points.

### Analysis of experimental results

#### Comparative analysis of models

To confirm the feasibility of our proposed hybrid strategy and the effectiveness of our model, we designed a series of comparative experiments. We compared the performance of different models in practical applications. Specifically, on the selected dataset, each model was independently run 30 times to ensure robustness of the results. During these independent runs, intelligent algorithms performed 500 optimization iterations for the objective function. The experimental outcomes are presented in tabular form. They include the MAPE, RMSE, and R^2^. These metrics represent the aggregate performance of each model across the 30 independent trials. Additionally, we recorded the training and prediction times under the support of swarm intelligence algorithms. These temporal metrics reflect the average duration consumed by SVR and the combined CPU-GPU-SVR during each training and prediction process. Through these comprehensive assessments, our study thoroughly examines the performance and efficiency of the proposed model. The results are shown in Table 3.

**Table 3:** Model comparison results.

NAME	Type	PSO-SVR	PSO-CPU-GPU-SVR	GA-GPU-CPU-SVR	SSA-CPU-GPU-SVR
Air Quality UCI (**AQLU**)	RMSE	117.1172554	119.5051371	121.3709651	**107.2987567**
	MAPE	0.727211661	0.77590601	0.838535283	**0.53512185**
Number of samples: 9358	R^2^	0.811587484	0.803614543	0.784017429	**0.965734659**
Features:15	Training time(S)	**59.7126379**	819.21436	830.92572	882.23621
	Prediction time(S)	**0.0040145**	51.74979	55.67677	55.33871
Beijing PM2.5 (2010.1–2013.12) (**BP10-13**)	RMSE	99.94405737	99.77823658	101.711558	**90.93115912**
	MAPE	0.768740951	0.745833944	0.84145769	**0.495533196**
Number of samples: 33,097	R^2^	0.845127642	0.858989267	0.874936126	**0.881604629**
Features: 4	Training time(S)	699.9019165	**40.22424807**	48.15695	112.6958
	Prediction time(S)	31.45462	0.69412	**0.43196**	1.33513
Beijing PM2.5 (2010.1–2014.12) (**BP10-14**)	RMSE	85.01805747	81.06272915	94.15656338	**79.34496198**
	MAPE	0.815126936	0.724034151	0.967073541	**0.670985693**
Number of samples: 43,824	R^2^	0.841019119	**0.891019119**	0.632759029	0.848273628
Features: 4	Training time(S)	3143.083628	**221.98376**	360.05402	332.94141
	Prediction time(S)	59.6414	**5.18547**	9.48605	12.68896
Beijing Multi-Site Air-Quality Data (Two station) (**BMTWO**)	RMSE	124.3656027	**119.144273**	142.8779934	126.6027772
	MAPE	0.498755091	0.492129602	0.995516258	**0.480304394**
Number of samples: 70,128	R^2^	0.789373541	0.798955096	0.627841067	**0.835322851**
Features:12	Training time(S)	18,975.909	**552.45801**	584.11469	756.53461
	Prediction time(S)	753.26308	**8.935505101**	15.20976	13.8271
Beijing multi-site air-quality data (Three station) (**BMTHREE**)	RMSE	129.9497606	126.549063	139.95041	**126.4900048**
	MAPE	0.756056129	0.765945502	0.922870102	**0.744272799**
Number of samples: 92,866	R^2^	0.621026602	**0.646910913**	0.379344487	0.588808941
Features: 12	Training time(S)	21,669.9554	**1544.21289**	1654.083379	1965.743976
	Prediction time(S)	817.43277	**16.10387**	18.20595	24.54617
Beijing multi-site air-quality data (Five station) (**BMFIVE**)	RMSE	126.7694186	**123.2080758**	138.9794616	125.1581696
	MAPE	0.749653128	0.689342146	0.812384937	**0.62626942**
Number of samples: 162,904	R^2^	0.651504317	**0.710804574**	0.483811524	0.679421323
Features: 12	Training time(S)	94,830.56335	**3363.582**	3573.61249	3817.20215
	Prediction time(S)	1048.87949	**26.82419**	48.16409	60.506881

Significant values are in bold.

Through comparative analysis, this study reveals significant differences in prediction accuracy among various models. Specifically, within datasets AQLU, BP10-13, and BP10-14, the SSA-CPU-GPU-SVR model outperforms four control models in terms of RMSE, MAPE, and R^2^. Although the PSO-CPU-GPU-SVR model's prediction accuracy is slightly inferior to that of the SSA-CPU-GPU-SVR model, the gap is narrowing over time. Notably, in terms of training time efficiency, the PSO-SVR model leads significantly in training time efficiency, being 13.71, 13.92, and 14.77 times faster than the other three models, respectively. Further analysis reveals that models integrating intelligent algorithms with CPU-GPU heterogeneous parallel computing are 6.21 to 17.40 times faster than the PSO-SVR model in training time on datasets BP10-13 and BP10-14. Among these models, the PSO-CPU-GPU-SVR model exhibits the shortest training time. Additionally, on datasets BMTWO, BMTHREE, and BMFIVE, the PSO-CPU-GPU-SVR model surpasses both the PSO-SVR and GA-CPU-GPU-SVR models in terms of RMSE, MAPE, and R^2^. While its accuracy is slightly lower than that of the SSA-CPU-GPU-SVR model, the difference is not significant. Remarkably, the PSO-CPU-GPU-SVR model has a substantial advantage in training time, being 14.03 to 35.34 times faster than the PSO-SVR model.

**Figure 5:**
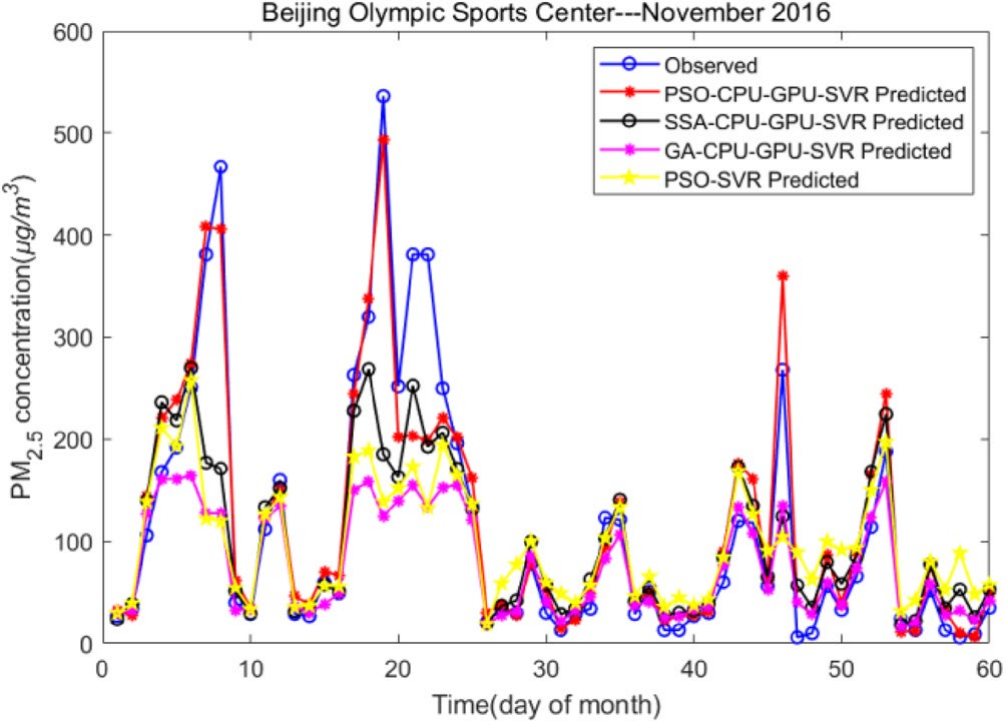
A comparison of the daily PM_2.5_ concentration predictions.

Based on the analysis provided, the PSO-CPU-GPU-SVR model exhibits exceptional prediction accuracy. Although it shows no significant difference in accuracy compared to the PSO-SVR model, it outperforms the GA-SVR model. Despite being slightly less accurate than the SSA-CPU-GPU-SVR model, it still meets the requirements for haze prediction in terms of overall performance. Hence, the PSO-CPU-GPU-SVR model offers a balance between prediction accuracy and computational efficiency, demonstrating its practicality and effectiveness in the field of haze prediction. To further illustrate the performance of the PSO-CPU-GPU-SVR model in practical applications, we selected meteorological data from the Beijing Olympic Sports Center area from November to December 2016. Further, the experiment juxtaposed the predicted values of each model with the actual values of the raw data to generate the fitted curves, as shown in Fig. 5.

As seen in Fig. 5, the predicted PM_2.5_ concentration values are consistent with the overall trend of the measured values. The prediction exhibits a minimal absolute error, which falls within the acceptable tolerance range. The model reflects the overall trend of PM_2.5_ concentration more sensitively. It also confirms the capability of the model to capture temporal changes effectively. Among all the compared models, the standardized value of PSO-CPU-GPU-SVR exhibited the highest error stability. The absolute deviation of the predicted value from the actual value is small, meaning that PSO-CPU-GPU-SVR can effectively cope with mutation data.

#### PSO-CPU-GPU asynchronous parallel SVR model performance analysis

The comparative analysis of models indicates that, on the AQLU dataset, the PSO-SVR model requires less time for both training and prediction than models combining CPU-GPU-SVR. Further investigation is conducted to determine the underlying reasons for this outcome.

**Figure 6:**
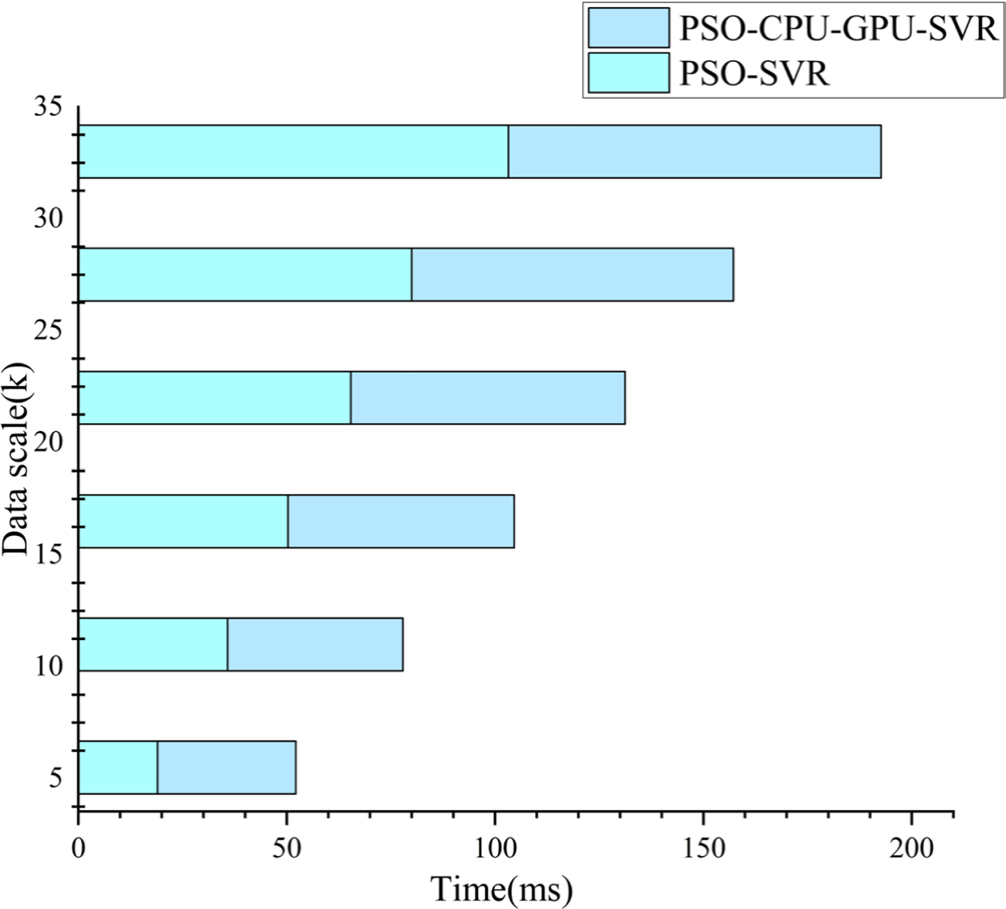
Comparison of PSO-CPU-GPU asynchronous parallel SVR model and PSO-SVR model runtime for small data sizes.

**Figure 7:**
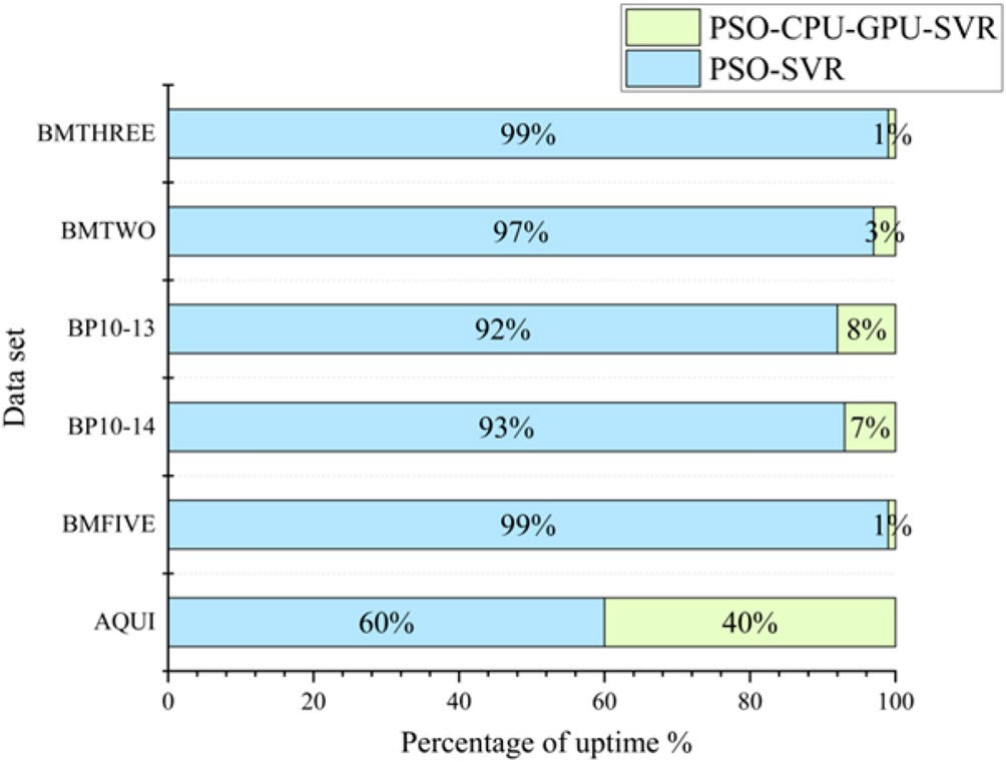
Comparison of PSO-CPU-GPU asynchronous parallel SVR model and PSO-SVR model runtime at large data sizes.

**Figure 8:**
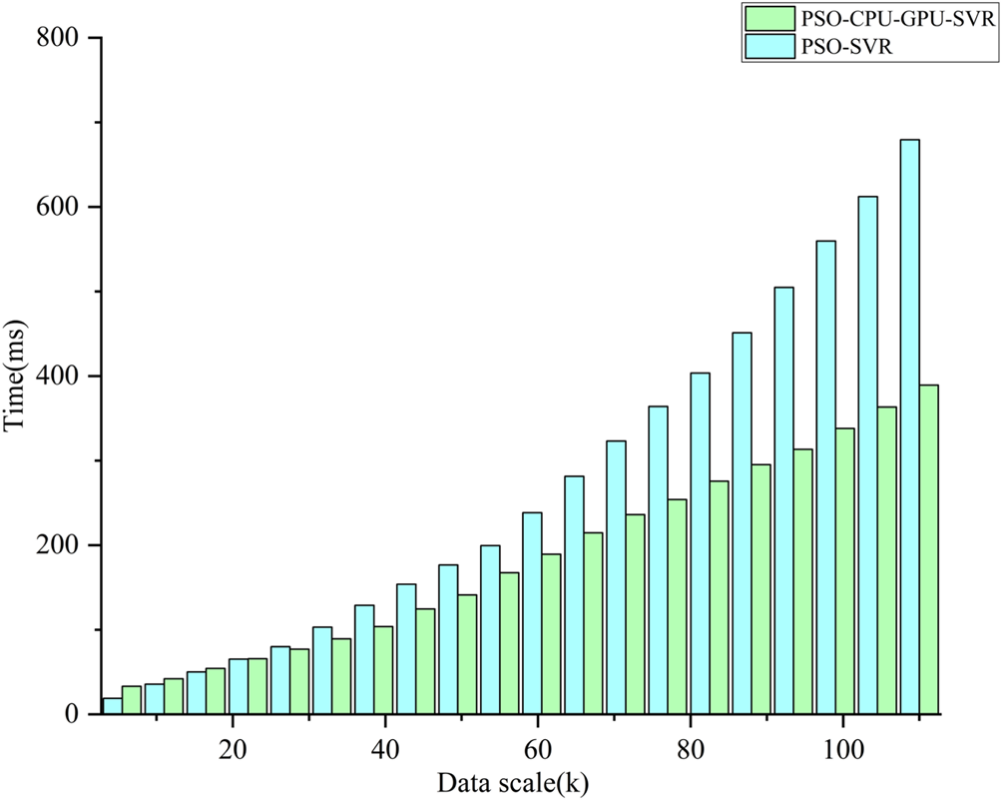
Data size and run time.

In CUDA, the data transfer bandwidth between the computer's main memory and GPU memory is much smaller than that of GPU memory. When processing small-scale data, the kernel program runs in a shorter time, while the inter-memory data transfer can take up a lot of time. As shown in Fig. 6, PSO-CPU-GPU asynchronous parallel SVR model has improved prediction accuracy for test samples as the scale of data increases. The former is a longer runtime than the latter. At large data sets, the PSO-CPU-GPU asynchronous parallel SVR model's computing time is significantly better than the PSO-SVR model's running time (see Fig. 7). This is because when the data size is relatively small. The data transfer time between the computer's main memory and GPU memory takes up a large portion of the time. This results in the PSO-CPU-GPU asynchronous parallel SVR model taking longer to operate at small data volumes than the PSO-SVR model. The proportion of data transfer time between the computer's main memory and GPU memory decreases gradually. As a result, the PSO-CPU-GPU asynchronous parallel SVR model is significantly more efficient. The operation efficiency of the PSO-CPU-GPU asynchronous parallel SVR algorithm increases significantly as the data scale increases. And the execution time of this algorithm increases less as shown in Fig. 8.

**Figure 9:**
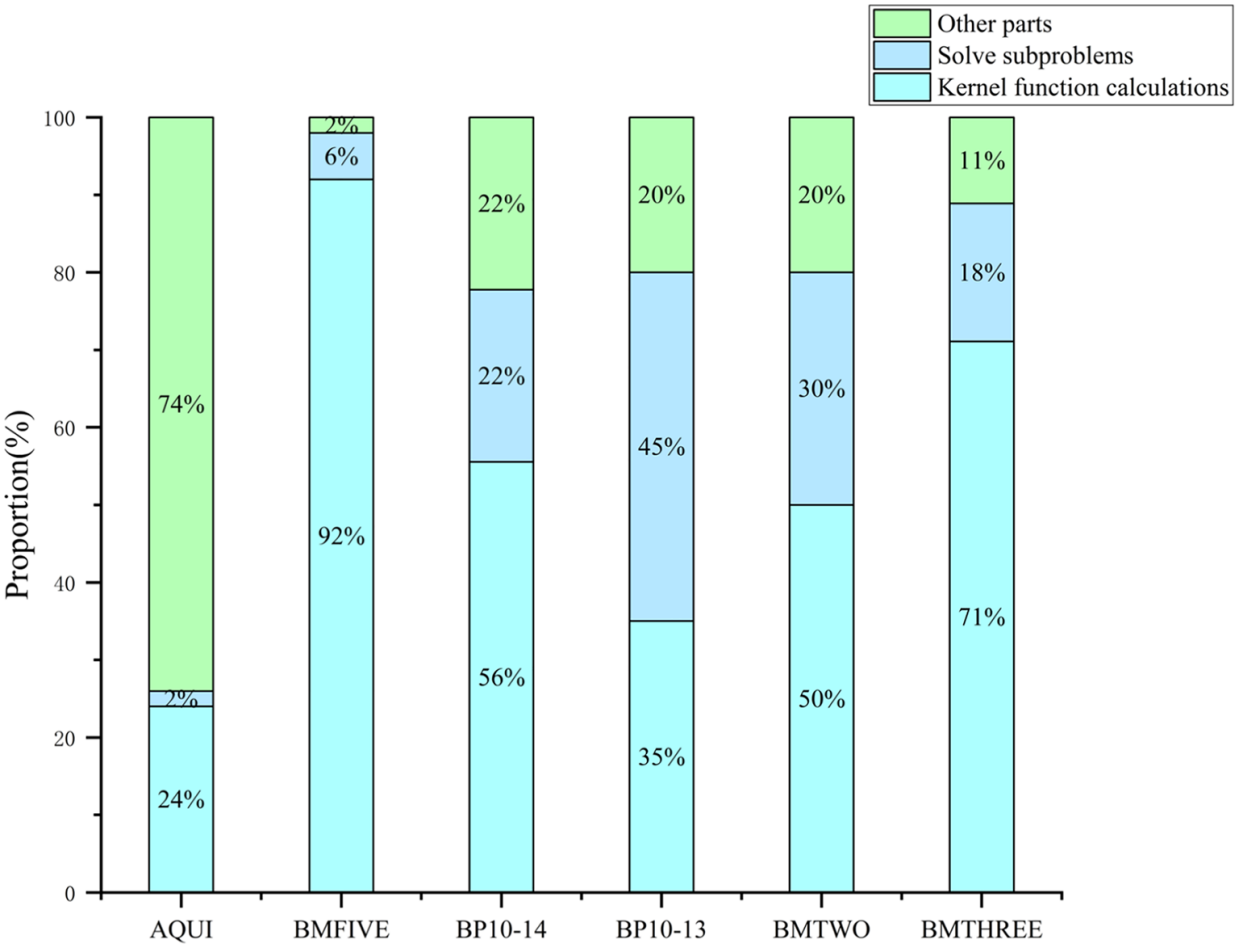
Time share of each component during training of PSO-CPU-GPU asynchronous parallel SVR models.

To demonstrate more clearly the effectiveness of the PSO-CPU-GPU asynchronous parallel SVR model and show its potential for improvement, this work measures the time taken by the key parts of the PSO-CPU-GPU asynchronous parallel SVR model to solve during training Percentage of the total time taken by each part of training as shown in Fig. 9. The main parts covered are the kernel function computation, solving the sub-problem (How to solve the sub-problem consisting of the working set.) and other parts that include operations such as selecting the working set and updating the parameters of the indicator function. The kernel function operations in the kernel call API take up most of the time in the specific process of intensive training.

## Conclusions

Accurate and timely PM_2.5_ concentration prediction is crucial for air quality management and public health. High-quality forecasts provide a scientific basis for air quality alerts and guide healthy public travel. Addressing this critical issue, our study introduces an efficient PM_2.5_ prediction model. The model is based on a high-efficiency code that we developed specifically for the SVR algorithm using the CUDA, which has significantly improved the execution efficiency of the algorithm. Building on this, we combine the PSO algorithm with SVR, capitalizing on the simplicity of the PSO structure, the convenience of parameter tuning, and its rapid convergence. This innovative integration optimizes computational efficiency and processing capacity of SVR in environmental data analysis. It also improves model performance by reducing data processing time, making it more suitable for large-scale datasets. Moreover, to validate the effectiveness of selecting PSO as our optimization algorithm, we performed separate assessments. We evaluated the PSO-CPU-GPU-SVR model against the GA-CPU-GPU-SVR and SSA-CPU-GPU-SVR models, respectively. Experimental results show that our heterogeneous parallel PSO-CPU-GPU-SVR model significantly outperforms in predicting PM_2.5_ concentrations. Compared to models integrating GA and SSA, PSO-CPU-GPU-SVR not only accelerates prediction speed but also exhibits better stability and reliability while maintaining high accuracy. These findings provide new insights into enhancing the efficiency and accuracy of air quality forecasting. They offer valuable data support for real-time air quality monitoring and decision-making. With reduced data processing time, our model is particularly suitable for large-scale environmental datasets, potentially advancing air quality prediction technology and informing other complex data analysis tasks.

Future research will focus on exploring more efficient algorithmic fusion strategies and parallel computing architectures, considering the growing scale and complexity of data. Additionally, addressing challenges encountered by PSO when handling large-scale data, such as optimization strategies and parameter tuning, warrants further investigation and improvement. Through continuous exploration and innovation, we aim to contribute more knowledge and technical solutions to the field of data-driven environmental monitoring and analysis.

## Data Availability

The datasets generated and analyzed during the current study are available from the corresponding author on reasonable request.

## References

[1] Chen ZY (2020). Influence of meteorological conditions on PM_2.5_ concentrations across China: A review of methodology and mechanism. Environ. Int..

[2] Bu X (2021). Global PM_2.5_-attributable health burden from 1990 to 2017: Estimates from the Global Burden of disease study 2017. Environ. Res..

[3] Lu XC (2019). Analysis of the adverse health effects of PM_2.5_ from 2001 to 2017 in China and the role of urbanization in aggravating the health burden. Sci. Total Environ..

[4] Liu ZH, Liu XH, Zhao KX (2023). Haze prediction method based on stacking learning. Stoch. Environ. Res. Risk Assess..

[5] Keerthi SS, Shevade SK, Bhattacharyy C, Murthy KRK (2001). Improvements to Platt's SMO algorithm for SVM classifier design. Neural Comput..

[6] Zhou QP, Jiang HY, Wang JZ, Zhou JL (2014). A hybrid model for PM_2.5_ forecasting based on ensemble empirical mode decomposition and a general regression neural network. Sci. Total Environ..

[7] Wiel KVD, Matthews AJ, Stevens DP, Joshi MM (2015). A dynamical framework for the origin of the diagonal South Pacific and South Atlantic Convergence Zones. Q. J. Roy. Meteorol. Soc..

[8] Cuomo S (2022). Scientific machine learning through physics-informed neural networks: Where we are and What's next. J. Sci. Comput..

[9] Han K (2023). A survey on vision transformer. IEEE Trans. Pattern Anal..

[10] Shaukat K, Luo SH, Varadharajan V (2023). A novel deep learning-based approach for malware detection. Eng. Appl. Artif. Intell..

[11] Castelli M, Clemente FM, Popovic A, Silva S, Vanneschi L (2020). A machine learning approach to predict air quality in California. Complexity.

[12] Balogun AL (2021). Spatial prediction of landslide susceptibility in western Serbia using hybrid support vector regression (SVR) with GWO, BAT and COA algorithms. Geosci. Front..

[13] Ikram RMA (2023). Advanced hybrid metaheuristic machine learning models application for reference crop evapotranspiration prediction. Agronomy-Basel.

[14] Karimipour A, Bagherzadeh SA, Taghipour A, Abdollahi A, Safaei MR (2019). A novel nonlinear regression model of SVR as a substitute for ANN to predict conductivity of MWCNT-CuO/water hybrid nanofluid based on empirical data. Phys A.

[15] Zhu SP, Keshtegar B, Ben Seghier ME, Zio E, Taylan O (2022). Hybrid and enhanced PSO: Novel first order reliability method-based hybrid intelligent approaches. Comput. Method Appl. Mech..

[16] Qi AL (2022). Directional mutation and crossover boosted ant colony optimization with application to COVID-19 X-ray image segmentation. Comput. Biol. Med..

[17] Li JQ, Liu ZM, Li CD, Zheng ZX (2021). Improved artificial immune system algorithm for Type-2 fuzzy flexible job shop scheduling problem. IEEE Trans. Fuzzy Syst..

[18] Kim J (2024). Development of an optimal post-processing model using the micro genetic algorithm to improve precipitation forecasting in Korea. Artif. Intell. Earth Syst..

[19] Feng ZK (2020). Monthly runoff time series prediction by variational mode decomposition and support vector machine based on quantum-behaved particle swarm optimization. J. Hydrol..

[20] Bezdan T (2021). Hybrid fruit-fly optimization algorithm with K-Means for text document clustering. Math-Basel.

[21] Mallick J (2022). Proposing receiver operating characteristic-based sensitivity analysis with introducing swarm optimized ensemble learning algorithms for groundwater potentiality modelling in Asir region, Saudi Arabia. Geocarto Int..

[22] Cao LJ, Keerthi SS, Ong CJ, Uvaraj P, Fu XJ, Lee HP (2006). Developing parallel sequential minimal optimization for fast training support vector machine. Neurocomputing.

[23] Glasmachers T, Igel C (2006). Maximum-gain working set selection for SVMs. J. Mach. Learn. Res..

[24] Matías J, Vaamonde A, Taboada J, González-Manteiga W (2004). Support vector machines and gradient boosting for graphical estimation of a slate deposit. Stoch. Environ. Res. Risk Assess..

[25] Norouzi H, Bazargan J, Taheri S, Karimipour A (2023). Investigation of unsteady non-Darcy flow through rockfill material using Saint–Venant equations and particle swarm optimization (PSO) algorithm. Stoch. Environ. Res. Risk Assess..

[26] Chang CC, Lin CJ (2011). LIBSVM: A library for support vector machines. ACM Trans. Intell. Syst. Technol..

[27] Gruber LF, West M (2016). GPU-accelerated Bayesian learning and forecasting in simultaneous graphical dynamic linear models. Bayesian Anal..

[28] Nickolls J, Dally WJ (2010). The GPU computing era. IEEE Micro.

